# Racial/Ethnic Residential Segregation and the First Wave of SARS-CoV-2 Infection Rates: A Spatial Analysis of Four U.S. Cities

**DOI:** 10.1177/07311214211041967

**Published:** 2021-10

**Authors:** Kathryn Freeman Anderson, Angelica Lopez, Dylan Simburger

**Affiliations:** 1 University of Houston, Houston, TX, USA; 2 Arizona State University, Phoenix, AZ, USA; 3 University of Arizona, Tucson, AZ, USA

**Keywords:** residential segregation, SARS-CoV-2, COVID-19, race/ethnicity, health inequality

## Abstract

Previous research has linked racial/ethnic residential segregation to a number of poor health conditions, including infectious disease. Here, we examine how racial/ethnic residential segregation is related to the novel coronavirus, SARS-CoV-2. We examine infection rates by zip code level segregation in four major cities across the U.S.: New York City, Chicago, Houston, and San Diego. We also include a number of area-level Census variables in order to analyze how other factors may help account for the infection rate. We find that both Black and Latino residential clustering are significantly and positively related to a higher SARS-CoV-2 infection rate across all four cities, and that this effect is strong even when accounting for a number of other social conditions and factors that are salient to the transmission of infectious disease. As a result, we argue that neighborhood-level racial/ethnic patterning may serve as an important structural mechanism for disparities in SARS-CoV-2 infection.

## Introduction

Racial/ethnic residential segregation has been linked to a number of social problems, and health inequity is no exception (Williams and Collins 2001). Previous work has demonstrated that segregation is related to higher rates of mortality, including infant mortality, overall poorer self-rated health, higher rates of obesity and poor nutrition quality, and a higher likelihood of many chronic health conditions (Collins and Williams 1999; Grady 2006; Subramanian, Acevedo-Garcia, and Osypuk 2005; Wen and Maloney 2011). This work has also shown that residential segregation is related to higher transmission of certain infectious diseases, which have strong ties to living conditions and poverty (Acevedo-Garcia 2000, 2001). Here, we propose to examine racial/ethnic residential segregation and the novel coronavirus SARS-CoV-2 (severe acute respiratory syndrome coronavirus 2), which causes coronavirus disease 2019 (COVID-19).

The news media has featured headlines on the disproportionate impact of the novel coronavirus on the black and Latino populations of the United States, but it is unclear how this is patterned by segregation (Centers for Disease Control and Prevention [CDC] 2020b). Scholarship on residential segregation has long demonstrated several adverse impacts of these residential configurations (Charles 2003). This work has referred to segregation as the “structural linchpin” of racial inequality in the United States, in that many other forms of racial inequality are directly related to the opportunity structures and exposures surrounding residence (Charles 2003; Massey 2020; Massey and Denton 1993). We posit that segregation may be positively related to transmission of the novel coronavirus, which may ultimately be related to higher rates of infection in these areas. We argue that this might be the case because of the substandard living conditions typical of segregated communities, such as crowding and poor housing quality (Acevedo-Garcia 2000). Moreover, these communities may also be more socially vulnerable and less able to practice social distancing because of the need to work in essential industries, working longer hours, and having fewer opportunities to work from home (Selden and Berdahl 2020; Tai et al. 2021; Williams and Collins 2001; Zheng et al. 2020).

With this associational study, we address two primary research questions.

**Research Question 1:** How is racial/ethnic residential segregation related to the first wave of SARS-CoV-2 coronavirus infections?
**Research Question 2:** What other aspects of neighborhoods are related to the infection rate, and could these help account for the higher infection rate?

We propose to address these questions in a series of analyses on four U.S. cities using zip-code-level data from local health departments and sociodemographic data from the American Community Survey (ACS). We specifically focus exclusively on the first wave of the pandemic in spring 2020 as a conservative test of these associations when many locales had fairly strict public health measures in place. We first detail the theoretical perspectives and prior work on segregation and the transmission of infectious disease.

## Theoretical Framework and Literature Review

### Segregation, Race, and Health

Extensive scholarship has documented the ill effects that racial/ethnic residential segregation can have on racial and ethnic minorities, particularly black segregation. Research presents theoretical and empirical evidence showing how discriminatory housing practices resulted in the concentration of racial and ethnic minorities in poor neighborhoods characterized by substandard housing and amenities (Charles 2003; Massey and Denton 1993; Massey, Gross, and Eggers 1991). Furthermore, opportunity structures and many social institutions in these areas have eroded, with residence associated with several adverse social outcomes (Charles 2003; Wilson 1987, 1996). As a result, scholars argue that racial/ethnic residential segregation creates areas that produce and maintain racial inequality in the United States. Through multiple forms of discrimination, racial/ethnic minority groups have differential experiences with U.S. opportunity structures and pathways to attaining residence relative to whites, which affords them limited locational mobility and attainment (Alba and Logan 1993; Charles 2003; Iceland and Wilkes 2006; Logan 1978). “Structural linchpin” of racial inequality, in that the mechanisms and adverse conditions that drive racial inequality in the United States are directly related to the historical process of segregation and the adverse conditions that accompany it (Charles 2003; Massey 2020). A now large body of literature has extended this line of thinking into the arena of health and has examined how segregation negatively impacts the health of residents.

Scholarship in this tradition finds that the health of residents living in segregated areas is adversely affected in many ways. Segregation has been linked to higher mortality (Collins and Williams 1999; Kramer and Hogue 2009), higher rates of infant mortality and low-birth-weight births (Grady 2006; Hearst, Oakes, and Johnson 2008; Walton 2009), and higher rates of preterm birth (Mason et al. 2011). Segregation is also associated with other poor health outcomes, such as higher rates of obesity (Chang, Hillier, and Mehta 2009; Ryabov 2018), lower rates of exercise (Mellerson et al. 2010), and higher rates of overall poor health reporting (K. F. Anderson 2016; Gibbons and Yang 2014; Subramanian et al. 2005). More theoretically, Williams and Collins (2001) frame segregation as a “fundamental cause” of racial health disparities in the United States, meaning that segregation is related to poor health outcomes via multiple mechanisms and for multiple disease outcomes. We posit that the current pandemic may be yet another case of these processes.

Moreover, Williams and Collins (2001) primarily discuss black-white segregation in their argument and are less clear on how segregation may pattern the health outcomes of other racial and ethnic minorities. While there are ambiguous associations between Asian segregation and health (Acevedo-Garcia et al. 2003; K. F. Anderson 2016; Walton 2012), research demonstrates that Latino segregation is increasing (Iceland 2004; Logan, Stults, and Farley 2004), and that Latino segregation more closely mirrors black segregation and often has negative health consequences for residents (Hall and Stringfield 2014; Mellerson et al. 2010; Wen and Maloney 2011). Here, we consider how black and Latino segregation specifically is related to SARS-CoV-2 infection rates.

Based on Williams and Collins’s (2001) fundamental cause argument, as well as empirical research examining how segregation impacts health outcomes, it is evident that segregation negatively patterns the health outcomes of racial and ethnic minorities in many ways. Based on this, it stands to reason that residents in segregated areas may be at greater risk of contracting infectious diseases as well, as research shows conditions of segregated areas may be correlated with conditions and lack of protections that encourage infectious disease transmission (Acevedo-Garcia 2000). Over the course of the COVID-19 pandemic, researchers and reporters have documented that blacks and Latinos are disproportionately impacted by SARS-CoV-2 transmission, COVID-19 deaths, and the economic fallout associated with the pandemic (CDC 2020b; Cerullo 2020; Krogstad, Gonzalez-Barrera, and Lopez 2020; National Urban League 2020; Tai et al. 2021; Thorbecke 2020; Yu et al. 2021). We argue that segregation may pattern the transmission of the SARS-CoV-2 coronavirus, as the substandard housing and poor economic conditions of segregated areas may encourage communicability of SARS-CoV-2. To elucidate this argument, we turn to the literature on residential segregation and infectious disease specifically.

### Segregation and Infectious Disease

Infectious disease is a broad term that encompasses a multitude of diseases caused by viruses, bacteria, or parasites (Morse 1995). Research indicates that infectious diseases are communicable illnesses that are spread through

skin contact, inhalation of airborne microbes, ingestion of contaminated food or water, bites from vectors such as ticks or mosquitoes that carry and transmit organisms, sexual contact [or] transmission from mothers to their unborn children via the birth canal and placenta. (Infectious Diseases Society of America 2020)

For this reason, many infectious diseases, including those that are respiratory in nature, are highly related to the social conditions in which people are situated.

Several studies on the spread of infectious diseases have pinpointed various social factors that exacerbate their spread (Hotez 2008; Krieger and Higgins 2002; Yousey-Hindes and Hadler 2011). These factors include more traditional public health variables, such as higher population density (Rocklöv et al. 2020) and poorer housing quality (Krieger and Higgins 2002), as well as more sociological variables such as lower socioeconomic status (Holtgrave and Crosby 2020; Hotez 2008) and restricted social networks (Adimora and Schoenbach 2005; Morris 1993; Mossong et al. 2008). For example, Zolopa and colleagues (1994) examined the spread of HIV and tuberculosis among the homeless population in San Francisco and found overcrowded homeless shelters and single occupancy hotel rooms to be associated with a higher rate of tuberculosis infections. Moreover, individuals from low socioeconomic backgrounds are more likely to reside in inadequate housing conditions. Krieger and Higgins (2002) note that conditions, such as pest problems and overcrowding, may result in an increase in inhabitants’ exposure to diseases carried by insects and rodents, and in an increase in the transmission rate of respiratory diseases within the household. Similarly, another study found that children living in high-poverty census tracts were three times more likely to be hospitalized for the flu than children in low-poverty census tracts (Yousey-Hindes and Hadler 2011). The researchers not only attribute this relationship to overcrowded housing conditions, but they also suggest that low socioeconomic status restricts individuals’ access to adequate health care to treat preexisting conditions that may lead infectious diseases to require hospitalization (Yousey-Hindes and Hadler 2011). There is also evidence that service sector jobs and public transportation expose individuals to higher rates of contagion (Baker et al. 2020). Furthermore, researchers have found that various forms of nonprivate transportation like air travel, buses, and trains facilitate the spread of infectious diseases (Edelson and Phypers 2011). For example, Feske and colleagues (2011) found a higher rate of endemic tuberculosis clustered strains among individuals who rode the bus at least once a week compared with nonbus riders. Another study found an association between the average time spent in buses and positive outcomes of tuberculosis (Edelson and Phypers 2011). Researchers suggest that the transmission of tuberculosis in buses and trains may be because they are enclosed spaces with poor ventilation (Edelson and Phypers 2011; Feske et al. 2011).

Moreover, individuals with preexisting comorbidities, such as HIV and diabetes, are at greater risk for infectious diseases as these conditions can weaken the immune system and put one at a greater risk of contraction (Feske et al. 2011; Shah and Hux 2003). For instance, a study on the diabetic population living in Ontario, Canada, found that almost half of the population (46 percent) had visited a physician or been hospitalized because of an infectious disease (Shah and Hux 2003). Other conditions such as obesity, hypertension, or old age may result in severe health complications from infectious diseases that are mild or symptomless to others (Fang 2020; Richardon 2020).

As it relates to segregation more specifically, an extensive literature has demonstrated strong positive associations between racial/ethnic segregation and rates of tuberculosis (Acevedo-Garcia 2000, 2001), sexually transmitted diseases (Adimora and Schoenbach 2005; Biello et al. 2012; Laumann and Yoosik 1999), and other infectious diseases (Hotez 2008; Strully 2011). Spatially isolated communities of racial and ethnic minorities with interrelated disadvantageous socioeconomic conditions often result in an increased risk of encountering infectious diseases and transmitting these diseases to others in the community (Acevedo-Garcia 2000). The associated characteristics of segregated communities, such as low education and income, limit the measures that its members can take to lower their risk of infection and lead to a dense cluster of infections in the community (Acevedo-Garcia 2001; Strully 2011). We argue that this can primarily occur through three primary pathways: socioeconomic resources, housing conditions and other place effects, and access to resources.

First, segregated communities are more likely to have characteristics of low socioeconomic status such as poverty, low educational attainment, inadequate housing conditions, and limited job opportunities. The interplay of these conditions further heightens the risk of people living in segregated communities of contracting an infectious disease and spreading it to others (Acevedo-Garcia 2001). Low educational attainment is associated with limited job opportunities that restrict individuals to service sector jobs and work that requires long hours that exposes them to communicable diseases and limit their freedom to take sick leave when needed (Hawkins 2020; Tai et al. 2021; Thorbecke 2020). For example, in one study, Buot and colleagues (2014) find that high transmission rates of HIV are associated with three factors found in segregated communities: income inequality, poverty, and low educational attainment.

Relatedly, due to the discriminatory processes of redlining and housing discrimination, racial/ethnic segregation is associated with a typically poorer and older housing stock. As such, these areas are more likely to have dilapidated housing conditions, such as older homes that expose inhabitants to mold and lead (Sampson and Winter 2016), overcrowded living conditions that facilitate the spread of respiratory diseases (Yousey-Hindes and Hadler 2011; Zolopa et al. 1994), and pest infested homes that lead to exposure to rodents and insects that spread infectious diseases (Krieger and Higgins 2002). Furthermore, segregated communities are more likely to be in environmentally hazardous areas, which may exacerbate respiratory illness (Bullard 2005; Frontera et al. 2020). For example, empirical research has shown that high-poverty minority children residing in segregated areas have higher rates of childhood asthma and are three times more likely to be hospitalized because of severe complications from the flu (Payne-Sturges and Gee 2006; Yousey-Hindes and Hadler 2011).

Finally, segregated communities have more limited access to jobs within close proximity of their residence, and because of diminished socioeconomic resources, individuals are more likely to rely on public transportation on a regular basis to go to work or access resources outside their community (M. Anderson 2016; Galaskiewicz, Anderson, and Thompson-Dyck 2021; Kain 1992). For instance, a study on the incidence of tuberculosis among individuals who use public transportation in the city of Houston found the duration of bus rides to be correlated to large cluster strains of tuberculosis (Feske et al. 2011). The findings of this study suggest that an increased distance of jobs and a wide variety of daily services from segregated communities result in an increased risk of catching a communicable disease.

### Segregation and the Case of SARS-CoV-2

COVID-19, specifically, is a respiratory infectious disease caused by the SARS-CoV-2 coronavirus, which is transmitted from person to person through the release of water droplets from infected individuals when coughing, sneezing, or talking (Bai et al. 2020; CDC 2020c). Infection can occur through direct contact with someone who has COVID-19 through the inhalation of respiratory droplets from an infected individual or, although somewhat less common, by touching surfaces which have been infected by those droplets (CDC 2020a). Because of the methods of transmission, the spread of COVID-19 is facilitated by close proximity and interaction between individuals, especially in indoor settings (CDC 2020a). Once initial contact with the virus occurs, an infected person might start exhibiting symptoms of COVID-19 within 2 to 14 days. On average, COVID-19 symptoms are mild and consist of fever, cough, shortness of breath, fatigue, body aches, headache, loss of taste or smell, sore throat, congestion, nausea, vomiting, and diarrhea (CDC 2020c). However, the severity of symptoms can vary. Some infected individuals are reported to be asymptomatic, while others develop severe complications such as ground-glass opacities, pneumonia, acute respiratory distress syndrome, and acute cardiac injury (Rothan and Byrareddy 2020). Notably, data suggest that individuals with certain preexisting conditions—old age, cardiac disease, hypertension, diabetes, and obesity—are at a higher risk of developing severe complications (Fang 2020; Richardon 2020). Regardless of the severity of symptoms, infected individuals can transmit COVID-19, even if asymptomatic (Bai et al. 2020). While vaccines, effective treatment plans, and better information on COVID-19 have been released, there was no comprehensive U.S. Food and Drug Administration (FDA)-approved pharmaceutical treatment for COVID-19 nor an effective prophylactic vaccine during the initial months of the pandemic, which is the study period here.

When considered together, many features of racial/ethnic segregated areas may encourage communicability of COVID-19 as noted above with our broader discussion of infectious disease. First, these areas are typically more densely populated, with large populations living in physically small areas or crowded housing units (Acevedo-Garcia 2000, 2001). In addition, impoverished, segregated areas likely produce arrangements where residents must rely more on public transportation and essential service work, make subpar wages, are unable to work from home, and may be unemployed (CDC 2020b; Williams and Collins 2001; Wilson 1996). The combination of these factors likely exacerbates the transmission of COVID-19 in segregated areas. Existing literature on employment and COVID-19 indicates that racial minorities are more likely to be employed in sectors with increased exposure to infection and decreased ability to work from home compared with whites (Benfer and Wiley 2020; Hawkins 2020; Kim and Bostwick 2020; Rogers et al. 2020; Selden and Berdahl 2020; Tai et al. 2021; Zheng et al. 2020). For instance, a study found a disproportionally higher percentage of blacks working in cleaning and maintenance, health care, transportation, food preparation, and personal care and service (Rogers et al. 2020).

In addition, several recent studies on the spread of COVID-19 analyzing residential segregation specifically also point to racial segregation as a contributor to the discrepancy of infection rates among racial and ethnic minorities (Khanijahani and Tomassoni 2021; Tan, deSouza, and Raifman 2020; Yang, Choi, and Sun 2021; Yu et al. 2021). For instance, Yu and colleagues (2021) found black and Latino segregated areas to have a faster growth rate of COVID-19 infections and deaths. Similarly, another study found white majority counties to have fewer COVID-19 infections than counties that are not predominately non-Latino white (Millet et al. 2020). Another study, also measured at the county level, also found that black and Latino population size was related to infection rates, and that this association was strengthened where the segregation of these groups from whites was higher (Yang et al. 2021).

Related black and Latino segregation, another consideration that some scholars theorize may be important to SARS-CoV-2 transmission specifically, is immigration as the lower socioeconomic profiles, precarious legal status, employment status, and living conditions of many foreign-born residents put them at higher risk of infection (Acevedo-Garcia 2000; Castaneda et al. 2015; Hall and Stringfield 2014). In contrast, the immigrant health paradox states that U.S. immigrants, despite their lower socioeconomic status and limited access to resources, have lower rates of mortality and better outcomes for certain diseases in relation to U.S. whites (Dubowitz, Barea, and Acevedo-Garcia 2010; Osypuk, Bates, and Acevedo-Garcia 2010; Riosmena, Kuhn, and Jochem 2017). The mechanisms researchers posit that motivate immigrant health are better social networks, relationships, and opportunity structures in their residential areas, in contrast to involuntary segregated areas that are more deprived from the discriminatory processes at play (Diaz, Koning, and Martinez-Donate 2016; Dubowitz et al. 2010). Consequently, based on this paradox, segregated areas with a high concentration of immigrants may be better protected from the transmission of infectious disease than other segregated communities and exhibit more protective health behaviors to protect against the transmission of infectious disease. However, the bolstered social relationships and sense of community in immigrant enclaves that are theorized to be beneficial to health may encourage SARS-CoV-2 transmission, as research suggests that communities that are more socioeconomically connected may exacerbate COVID-19 communicability (Hamida, Sabouri, and Ewing 2020). Furthermore, immigrant communities with limited socioeconomic resources still possess several characteristics like high population density and subpar housing that encourage the transmission of infectious diseases and likely COVID-19 as well (Osypuk et al. 2010; Osypuk et al. 2009).

Due to the relationships discussed above, we argue that segregation may increase the communicability of the SARS-CoV-2 virus for blacks and Latinos. Segregation is associated with a number of social risk factors for both blacks and Latinos that research finds are associated with increased transmission of the SARS-CoV-2 coronavirus. Based on the above discussion of how segregation may pattern the transmission of SARS-CoV-2 for blacks and Latinos, we formulate the following two hypotheses:

**Hypothesis 1:** An increase in black and Latino segregation across areas will be related to an increase in the SARS-CoV-2 infection rate.
**Hypothesis 2:** Social risk factors associated with segregation will help explain the relationship between black/Latino segregation and the SARS-CoV-2 infection rate across areas.

To test these hypotheses, we present a study analyzing the cross-sectional association between segregation, the infection rate across neighborhoods, and social risk factors for four large cities in the United States. We detail our data and methodological approach below.

## Data and Methods

### Data and Variables

To answer these research questions, we combined several primary data sources, all measured at the zip code tabulation area (ZCTA) unit of analysis. For this analysis, we use data on infections at the zip code level for four major urban areas in the United States: New York City, Chicago, Houston, and San Diego. When originally imagining this project, we intended to collect data for as many large U.S. cities as possible to combine them into a single model, but due to issues of data comparability and availability, this was not possible. There have been vast differences in testing capabilities, distribution of testing facilities, and reporting of positivity and testing rates across public health jurisdictions making it difficult to compare infection rates across areas. Given these differences, putting all of the infection rates across areas into a single model may just reflect differences in the testing apparatus and reporting rather than differences in infection rates. Furthermore, most locations have not provided data on testing and positivity rates, which could serve as critical controls for these problems. In our data, only New York City and Chicago provide data on the number of tests administered in a particular zip code. We do not include these in the models presented here as we wanted the models to be comparable across all areas. However, for these two cities at least, we ran all of the same models using the number of tests as a control variable to see whether this would change our conclusions. While the number of tests administered was in both cases significant and positively associated with the infection rate, their inclusion does not change the conclusions and the variables for racial/ethnic clustering (results available upon request). Therefore, we present an analysis for each urban area independently because the data for each area come from a single public health jurisdiction. We use these four urban areas as they span the range of the United States geographically with one urban area per each major Census region (Northeast, Midwest, South, and West), and they reflect some of the largest cities in the nation. New York, Chicago, and Houston are three of the four largest cities in the county, where San Diego is the eighth largest city and was the largest city for which data were available at the zip code level in the West.^1^

Furthermore, the data all come from dates in the middle of May (exact date depending on local data availability) and reflect cumulative infections for each zip code. While areas across the United States across the duration of the pandemic have experienced several waves of highs and lows in terms of infection rates, we chose this first wave time frame as a conservative test of our hypotheses. In May, some states began to open up nonessential facilities and allow nonessential travel, and as a result, starting in June, we begin to see divergent trends in the growth of the virus across the nation. This time frame also marks a growing politicization of the virus and policies around controlling its spread, such as mask-wearing and lockdown policies. Therefore, we use dates in mid-May to capture a conservative test of the viral spread during a time under which most states, counties, and cities were under some form of a stay-at-home order, and more aggressive efforts were being taken to control infection rates. Essentially, it is an examination of who was most vulnerable to the virus when the most protections were in place across the United States. After this period, we see a greater divergence in policies used to combat the virus with increasingly political stances on such measures across states.

The data for New York City come from NYC Health and reflect the cumulative count of positive tests across each of the zip codes in the five boroughs of the City of New York from May 13, 2020. The data for Chicago come from the Illinois Department of Public Health and reflect the cumulative count of positive tests from all zip codes in the Chicago metropolitan area that are geographically contained within Illinois (excludes those zip codes in Indiana) from May 11, 2020. The data for Houston come from the Houston Health Department and Harris County Public Health and reflect the cumulative count of positive test across all zip codes in both the City of Houston and Harris County (the core county of the Houston area) from May 23, 2020. The data for San Diego come from the County of San Diego Health and Human Services Agency and reflect the cumulative count of positive test from all zip codes in the County of San Diego (the core county of the City of San Diego) from May 22, 2020.^2^ For this analysis, to analyze the data using spatial methods, we converted this figure to the rate of infections per 10,000 people in the population. The *infection rate* is the dependent variable across all models.

We combine these data with the 2014–2018 ACS at the ZCTA level of analysis. We use the five-year estimates spanning the 2014 to 2018 range as this is the most recent year of publicly available data. The data are also combined into five-year estimates in this fashion to make the data representative at a small unit of analysis (like the zip code), as the ACS reflects a sample survey and is not representative at the zip code level for any given single year. We use several measures from the ACS as independent variables in the analysis to estimate the relationship between segregation and infections, as well as account for many other neighborhood-level factors that could contribute to the spread of the disease.

For this analysis, we use two primary variables from this source, including percent black and percent Latino to construct two segregation scores. Typically, segregation scores, like the popular indices of dissimilarity and isolation, are measured at a fairly large geographical unit of analysis like the metropolitan area or county to measure the distribution of groups across an urban area (Massey and Denton 1988). However, we are primarily interested in which subunits (in this case, zip codes) within an urban area would be characterized as minority segregated areas. In this case, global scores, like the index of dissimilarity or isolation, would not be appropriate, as they would indicate how different racial groups are spread out over a relatively small area, which is not the goal of this analysis, especially because due to residential segregation, many small geographic units have relatively homogeneous populations (Massey and Denton 1988). Often scholars in this tradition use racial/ethnic density scores, or the percent of a group over a defined area (Shaw and Pickett 2011).^3^ These types of scores are inherently aspatial, though, and fail to measure the geographical clustering of groups across spatial units of analysis. More recent scholarship in both sociology and geography alike have called for spatial approaches to the problem and have suggested multiple measures (Reardon and O’Sullivan 2004; Roberto 2018). To capture this, we construct two scores using these measures to account for geographic clustering in space by taking into account the group density in particular zip code (concentration) and also the extent to which physically adjacent zip codes have high numbers of the same group (clustering) (Anderson 2017). The formula we used is the following:

$$C_{i}=x_{i}\sum_{j=1,\;j\neq i}^{n}w_{ij}x_{j},$$

where $x_{i}$ is the variable for feature *i*, $x_{j}$ is the variable for feature *j*, and $w_{ij}$ is the spatial weight between features *i* and *j* (Anderson 2017). The formula reflects the product of the percent of a group in a zip code and the average percent (for row standardized weights) in its neighbors as indicated by a first-order queen contiguity matrix. As such, the measure can theoretically span 0–10,000, as it is the product of two numbers with a range of 100, but the upper bound of 10,000 is not empirically observed in any of our case cities. Thus, we include across all models two segregation clustering scores: *percent black clustering* and *percent Latino clustering*.^4^

Of note, the patterns in segregation across the United States vary considerably (including these four cities), and these measures are sensitive to the relative size of the group in an area. However, because each city is analyzed in separate model (details below), the results are still relative to that area, as opposed to in comparison with other locales across the United States. As a point of reference, for better understanding segregation across these areas, using the index of dissimilarity, which is the most commonly used global measure of segregation, New York City and Chicago, with scores of 76.89 and 75.15, respectively, are considerably more segregated for blacks than Houston and San Diego, with scores of 60.61 and 48.37 respectively. Across all locales, these figures are also lower for Latino-white dissimilarity at 62, 56.32, 52.51, and 49.61 for New York, Chicago, Houston, and San Diego, respectively. However, when using a global segregation measure that is more sensitive to the relative group sizes—the isolation index, for example—these scores are notably lower for areas with few members of that group. For example, the black isolation index in San Diego is only 11.24 (as compared with 51.33, 64.79, and 37.23 for New York City, Chicago, and Houston, respectively), as the black population of San Diego is quite small at only 5 percent. Thus, how we make sense of the results and these clustering scores should be situated within the demographics and global segregation levels of the area.

We also include several other covariates, which have been shown to be important to health generally or to the case of COVID-19 disease specifically (CDC 2020a; Williams and Collins 2001). These include immigration, as measured by *percent foreign born*, as some have theorized that immigrant enclaves are more likely to experience housing crowding. We also include a measure for the elderly population to capture this portion of the population that is more susceptible to the disease, which is measured by the *percent of people aged 65 and above*. We include several measures for social conditions, such as socioeconomic status with measures for *percent with a bachelor’s degree or above*, *percent in poverty*, and the *unemployment rate*, and household factors, as measured by *housing crowding* (measured by the percent of households with 1.5 persons per room or more), *median household income*, and *percent new residents* (measured by the percent of households that moved into the area since the last Census), as these may be indicators of mobility in and out of an area and thus possible exposure. We include several variables meant to capture how likely people are to be able to maintain social distancing, especially as it relates to work. These are measured by the *percent of people in the population who use public transportation for their normal work commute*, the *percent of people who work from home*, and the *percent of people in a professional industry*, the latter of which may serve as a proxy for the ability to work from home as the Census measure for the percent of people who work from home pre-dates the pandemic. Finally, we include several occupational variables for categories that may be more likely to be essential workers, including the *percent of people who work in service, sales, and production*. Descriptive statistics for each city are available upon request.

### Methods

**Figure 1.**
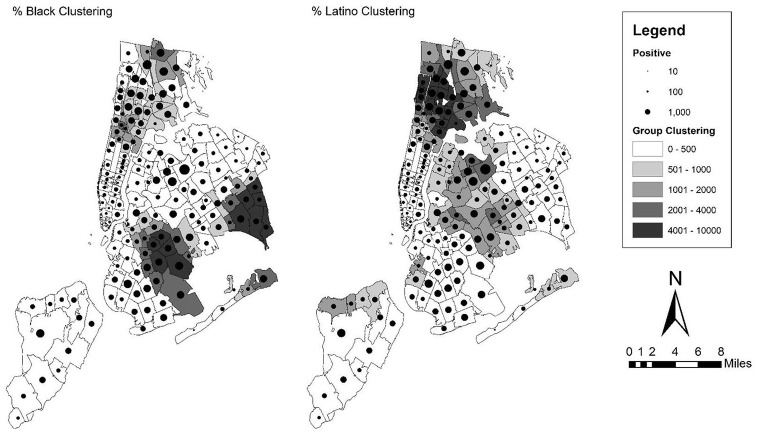
Map of SARS-CoV-2 infections over black and Latino clustering scores in zip code tabulation areas of New York City. *Note.* SARS-CoV-2 = severe acute respiratory syndrome coronavirus 2.

**Figure 2.**
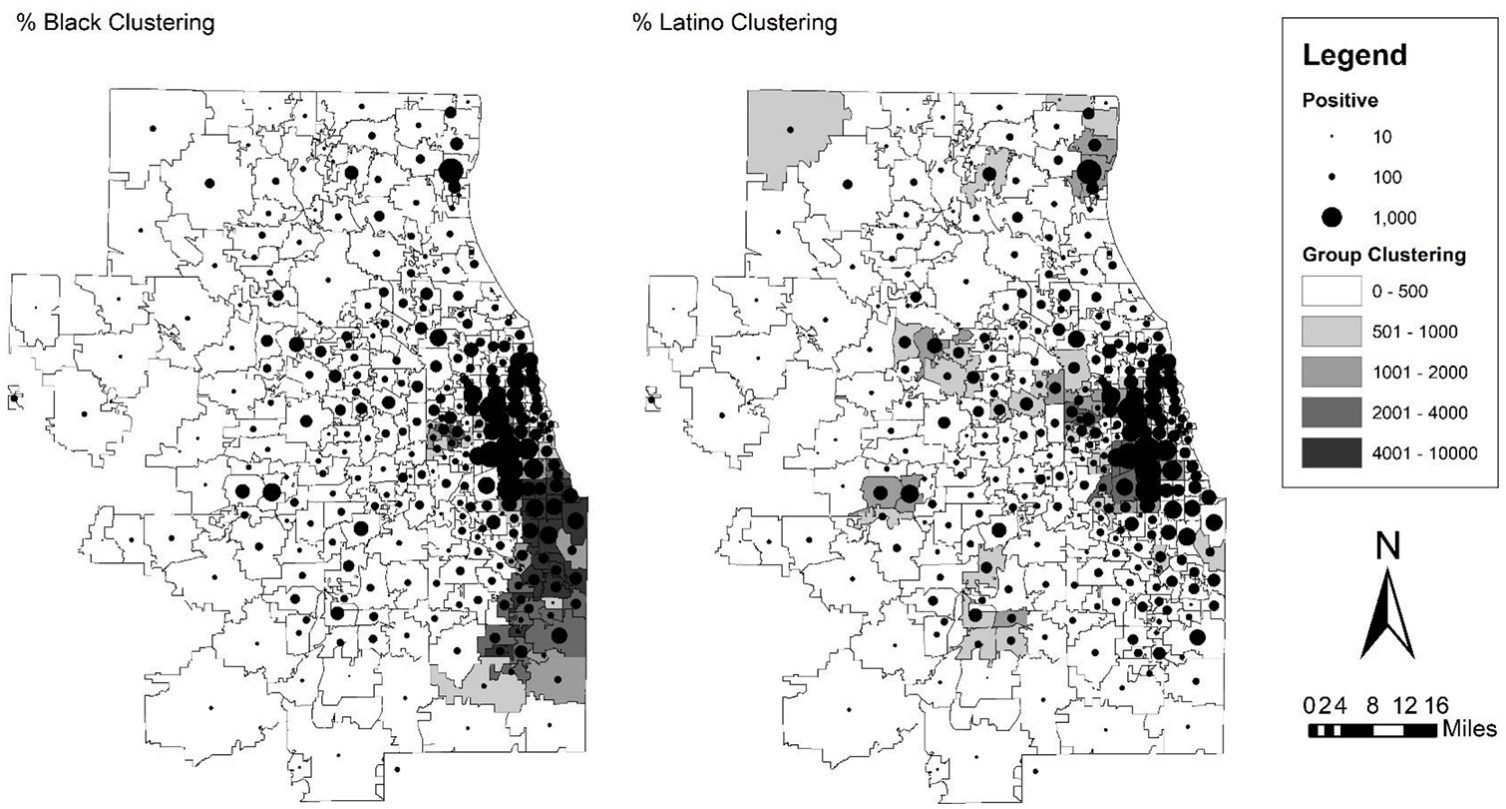
Map of SARS-CoV-2 infections over black and Latino clustering scores in zip code tabulation areas of Chicago. *Note.* SARS-CoV-2 = severe acute respiratory syndrome coronavirus 2.

**Figure 3.**
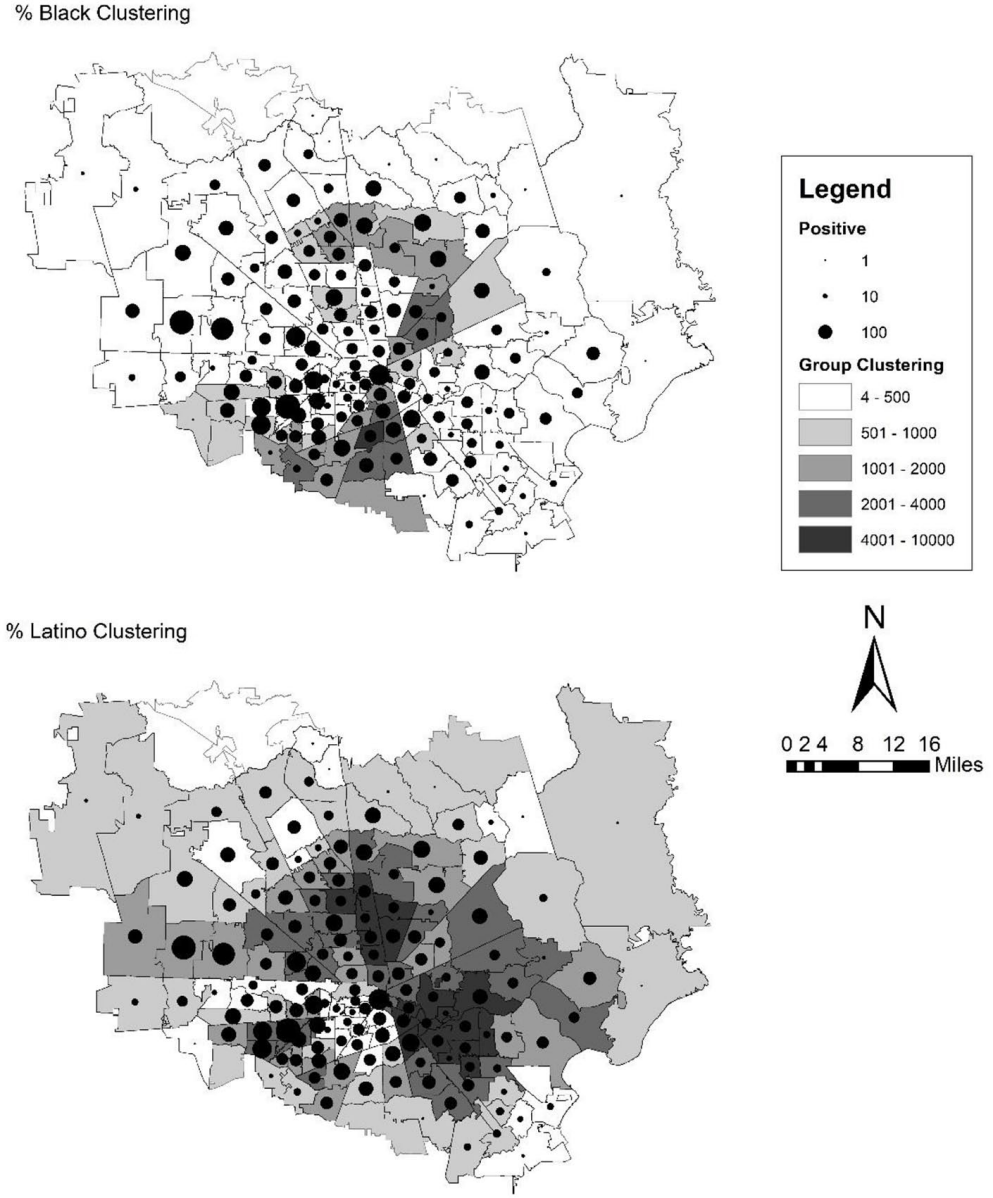
Map of SARS-CoV-2 infections over black and Latino clustering scores in zip code tabulation areas of Houston. *Note.* SARS-CoV-2 = severe acute respiratory syndrome coronavirus 2.

**Figure 4.**
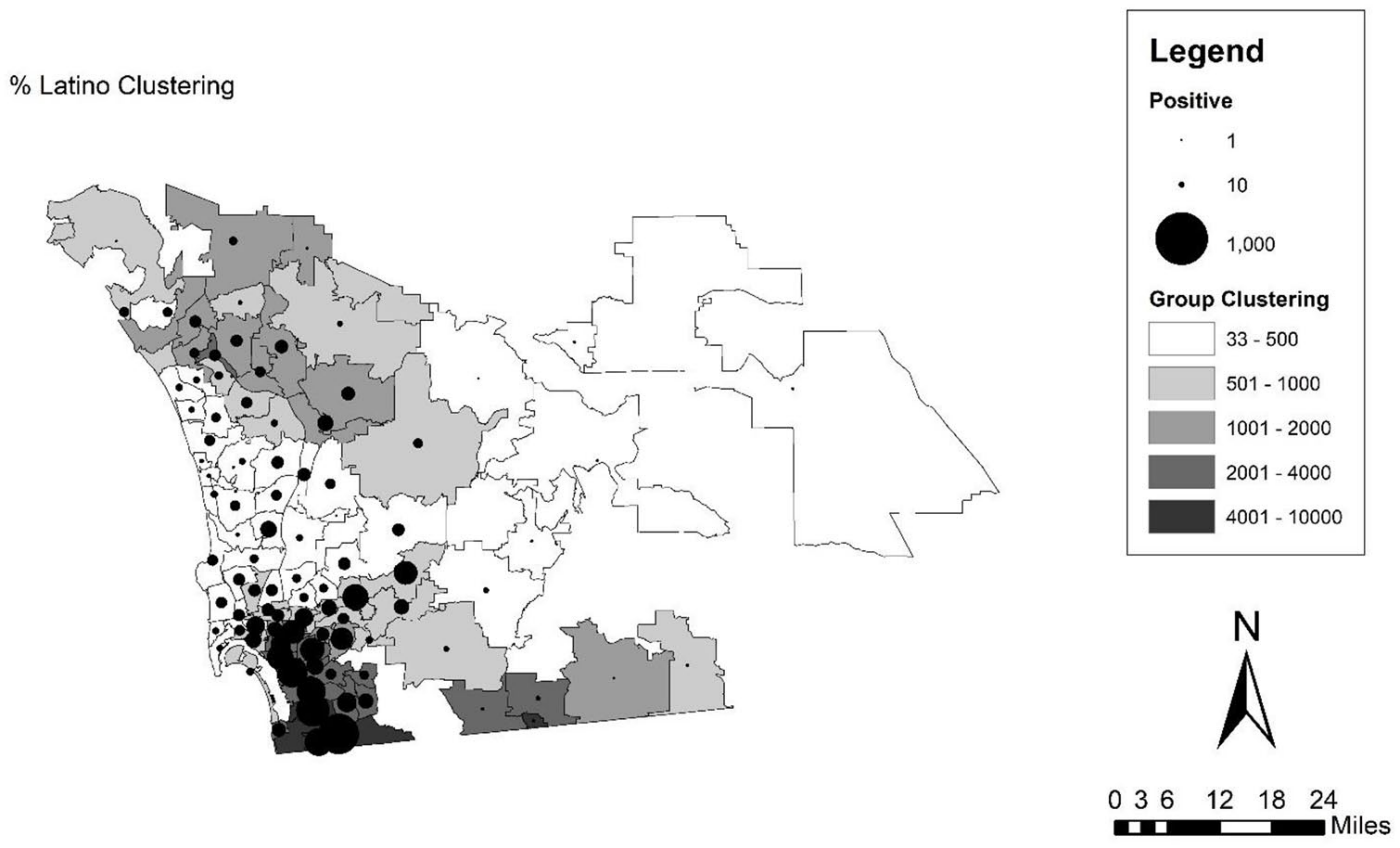
Map of SARS-CoV-2 infections over Latino clustering scores in zip code tabulation areas of San Diego. *Note.* SARS-CoV-2 = severe acute respiratory syndrome coronavirus 2.

For all models, we estimate a series of spatial error models to account for significant spatial autocorrelation between the geographic units of analysis, as physically proximate areas may influence each other, and therefore not meet the assumption of independence of error terms. The spatial error model specifically was found to be the most appropriate spatial model according to the Lagrange multiplier statistics, which are a set of statistics that indicate whether the source of spatial autocorrelation is principally driven by the errors, the dependent variable, or both (Anselin 1995; Anselin, Florax, and Rey 2004). In each model, we use a first-order queen contiguity spatial weight matrix (as compared with rook, several *k* nearest neighbor specifications, and geographic distance spatial weights), as it was found to best maximize Global Moran’s *I*, which is a measure of spatial autocorrelation, for our dependent and key independent variables, meaning that it accounts for the largest amount of spatial autocorrelation between units (Anselin 1995; Anselin et al. 2004). We present a series of models for each of our example cities. We begin with a model that just includes the racial/ethnic clustering scores and population density as a control. And, then we add groups of potentially explanatory variables to account for the various factors that have been proposed (both in the literature on segregation broadly and in the current pandemic) to explain away the relationship. As this analysis is meant to be exploratory by examining a wide variety of variables theorized to be related to the pandemic, we do not include formal mediation tests for the groups of variables included, and instead rely on this informal method by examining changes in the coefficients across model specifications. We present each set of models separately for the four urban areas in our study. Results for New York City can be found in Table 1, Chicago in Table 2, Houston in Table 3, and San Diego in Table 4. We also created a series of maps to visually display our findings as well (see Figures 1–4). We discuss our findings for each city in turn.

**Table 1.** Coefficients and (Standard Errors) for Variables Used in Spatial Error Models of SARS-CoV-2 Infections in New York City (*N* = 176).

Variable name	Race/ethnicity	Immigration	Age	SES	Household	Commute	Occupation	Full model
Black clustering	0.015** (0.005)	0.014** (0.004)	0.015*** (0.005)	0.006 (0.004)	0.010* (0.004)	0.009* (0.004)	0.008^†^ (0.004)	0.010* (0.004)
Latino clustering	0.030*** (0.006)	0.027*** (0.006)	0.032*** (0.006)	0.013* (0.006)	0.020*** (0.005)	0.025*** (0.006)	0.014* (0.007)	0.021*** (0.006)
Population density^a^	−0.550*** (0.139)	−0.574*** (0.139)	−0.542*** (0.138)	−0.320* (0.138)	−0.477*** (0.139)	−0.313* (0.141)	−0.388** (0.129)	−0.323* (0.140)
% Foreign born		0.766^†^ (0.412)						−0.212 (0.463)
% Age 65 and up			1.916* (0.789)					2.035* (0.998)
% Bachelor’s degree				−2.116*** (0.375)				−0.559 (0.831)
% in poverty				−0.565 (0.680)				−0.199 (0.787)
Unemployment rate				−2.332 (1.750)				−1.710 (1.755)
Housing crowding					5.436** (1.770)			4.474* (2.067)
Median home value^a^					−0.032* (0.014)			−0.005 (0.014)
% New residents					−2.119*** (0.427)			−0.255 (0.619)
% Public transportation						−1.008* (0.397)		−0.842^†^ (0.441)
% Work from home						−7.893*** (2.226)		−5.668* (2.465)
% Professional industry						−2.694* (1.056)		−0.504 (1.346)
% Sales							4.624*** (1.122)	2.381 (1.464)
% Service							1.193 (0.812)	0.315 (1.479)
% Production							4.219** (1.589)	1.571 (2.008)
Lambda	0.734*** (0.046)	0.717*** (0.047)	0.729*** (0.047)	0.647*** (0.058)	0.635*** (0.055)	0.606*** (0.063)	0.693*** (0.053)	0.637*** (0.062)
Pseudo-*R*^2^	.410	.445	.444	.616	.601	.660	.585	.683

*Note.* Data come from the 2014 to 2018 American Community Survey and the New York City Department of Health. SARS-CoV-2 = severe acute respiratory syndrome coronavirus 2; SES = socioeconomic status.

a The coefficient and standard error multiplied by 1,000 for the ease of presentation.

† *p* < .1. *p < .05. **p < .01. ***p < .001 (two-tailed).

**Table 2.** Coefficients and (Standard Errors) for Variables Used in Spatial Error Models of SARS-CoV-2 Infections in Chicago (*N* = 309).

Variable name	Race/ethnicity	Immigration	Age	SES	Household	Commute	Occupation	Full model
Black clustering	0.015*** (0.002)	0.018*** (0.002)	0.015*** (0.002)	0.003 (0.002)	0.013*** (0.002)	0.010*** (0.002)	0.010*** (0.002)	0.005* (0.002)
Latino clustering	0.041*** (0.004)	0.033*** (0.004)	0.042*** (0.004)	0.030*** (0.004)	0.035*** (0.004)	0.037*** (0.004)	0.029*** (0.004)	0.026*** (0.004)
Population density^a^	0.409 (0.470)	−0.187 (0.469)	0.669 (0.462)	0.359 (0.465)	−0.125 (0.529)	−0.470 (0.503)	1.055* (0.464)	−0.356 (0.507)
% Foreign born		1.251*** (0.249)						0.863*** (0.253)
% Age 65 and up			0.870** (0.291)					0.806* (0.341)
% Bachelor’s degree				−0.285* (0.144)				0.583^†^ (0.343)
% in poverty				1.732*** (0.355)				0.733^†^ (0.423)
Unemployment rate				2.293** (0.834)				1.875* (0.856)
Housing crowding					15.489*** (3.098)			7.716** (2.978)
Median home value^a^					−0.048** (0.017)			−0.026 (0.023)
% New residents					−0.436* (0.206)			−0.155 (0.230)
% Public transportation						1.383*** (0.238)		0.545^†^ (0.317)
% Work from home						−2.928*** (0.715)		−0.234 (0.757)
% Professional industry						−1.306* (0.518)		−0.338 (0.600)
% Sales							2.305*** (0.305)	1.264** (0.412)
% Service							1.240** (0.458)	1.213* (0.566)
% Production							1.295** (0.399)	1.239* (0.578)
Lambda	0.445*** (0.070)	0.450*** (0.071)	0.424*** (0.070)	0.538*** (0.054)	0.471*** (0.060)	0.512*** (0.059)	0.543*** (0.053)	0.460*** (0.063)
Pseudo-*R*^2^	.490	.529	.510	.591	.531	.548	.547	.676

*Note.* Data come from the 2014 to 2018 American Community Survey and the Illinois Department of Public Health. SARS-CoV-2 = severe acute respiratory syndrome coronavirus 2; SES = socioeconomic status.

a The coefficient and standard error were multiplied by 1,000 for the ease of presentation.

† *p* < .1. *p < .05. **p < .01. ***p < .001 (two-tailed).

**Table 3.** Coefficients and (Standard Errors) for Variables Used in Spatial Error Models of SARS-CoV-2 Infections in Houston (*N* = 153).

Variable name	Race/ethnicity	Immigration	Age	SES	Household	Commute	Occupation	Full model
Black clustering	0.009*** (0.002)	0.009*** (0.002)	0.009*** (0.002)	0.006** (0.002)	0.010*** (0.002)	0.006*** (0.002)	0.009*** (0.002)	0.007*** (0.002)
Latino clustering	0.001* (0.001)	0.001^†^ (0.001)	0.001 (0.001)	−0.000 (0.001)	0.002** (0.001)	0.002*** (0.001)	0.002^†^ (0.001)	−0.000 (0.001)
Population density^a^	2.098*** (0.493)	2.132*** (0.607)	2.041*** (0.492)	1.771** (0.576)	1.437** (0.551)	0.601 (0.566)	1.472* (0.620)	0.802 (0.615)
% Foreign born		−0.011 (0.166)						0.097 (0.145)
% Age 65 and up			−0.643^†^ (0.386)					−0.637** (0.397)
% Bachelor’s degree				0.011 (0.100)				−0.927*** (0.182)
% in poverty				0.517* (0.223)				0.012 (0.238)
Unemployment rate				0.121 (0.655)				0.298 (0.537)
Housing crowding					−0.372 (1.053)			−0.665 (1.017)
Median home value^a^					0.003 (0.010)			0.016 (0.011)
% New residents					0.353** (0.116)			0.224^†^ (0.122)
% Public transportation						3.090*** (0.627)		2.787*** (0.654)
% Work from home						0.947 (0.692)		0.944 (0.697)
% Professional industry						0.274 (0.394)		1.234** (0.458)
% Sales							−0.695^†^ (0.362)	−0.687* (0.344)
% Service							0.130 (0.284)	−1.158*** (0.344)
% Production							−0.339 (0.341)	−0.796* (0.404)
Lambda	0.047 (0.090)	0.044 (0.091)	0.054 (0.091)	−0.001 (0.096)	−0.071 (0.102)	−0.132 (0.111)	−0.026 (0.096)	−0.231 (0.155)
Pseudo-*R*^2^	.262	.263	.275	.294	.304	.373	.289	.511

*Note.* Data come from the 2014 to 2018 American Community Survey and the Houston Health Department and Harris County Public Health. SARS-CoV-2 = severe acute respiratory syndrome coronavirus 2; SES = socioeconomic status.

a The coefficient and standard error were multiplied by 1,000 for the ease of presentation.

† *p* < .1. *p < .05. **p < .01. ***p < .001 (two-tailed).

**Table 4.** Coefficients and (Standard Errors) for Variables Used in Spatial Error Models of SARS-CoV-2 Infections in San Diego (*N* = 97).

Variable name	Race/ethnicity	Immigration	Age	SES	Household	Commute	Occupation	Full model
Black clustering	0.028 (0.036)	0.012 (0.037)	0.027 (0.037)	0.002 (0.038)	−0.036 (0.034)	−0.020 (0.035)	0.046 (0.038)	−0.063* (0.030)
Latino clustering	0.009*** (0.001)	0.011*** (0.002)	0.009*** (0.001)	0.007*** (0.001)	0.006*** (0.001)	0.009*** (0.001)	0.008*** (0.002)	0.006*** (0.001)
Population density^a^	0.376 (0.516)	0.766 (0.537)	0.372 (0.518)	0.553 (0.505)	1.559** (0.514)	0.703 (0.550)	0.142 (0.539)	1.046* (0.498)
% Foreign born		−0.439* (0.178)						−0.270^†^ (0.150)
% Age 65 and up			−0.020 (0.198)					−0.722*** (0.185)
% Bachelor’s degree				−0.183* (0.091)				−0.192^†^ (0.114)
% in poverty				−0.281 (0.185)				−0.210 (0.182)
Unemployment rate				0.927* (0.431)				1.569*** (0.380)
Housing crowding					1.179 (0.772)			1.008 (0.811)
Median home value^a^					−0.009^†^ (0.005)			−0.006 (0.005)
% New residents					−0.465*** (0.099)			−0.318** (0.104)
% Public transportation						1.229 (0.767)		2.045* (0.835)
% Work from home						0.588*** (0.121)		0.443*** (0.129)
% Professional industry						0.079 (0.274)		0.593* (0.278)
% Sales							0.390 (0.242)	0.801*** (0.207)
% Service							0.164 (0.200)	−0.264 (0.218)
% Production							0.049 (0.440)	−0.696* (0.352)
Lambda	0.222* (0.106)	0.288* (0.114)	0.223* (0.106)	0.280** (0.086)	0.180^†^ (0.094)	0.124 (0.082)	0.249* (0.109)	−0.056 (0.101)
Pseudo-*R*^2^	.460	.483	.460	.500	.586	.571	.474	.755

*Note.* Data come from the 2014 to 2018 American Community Survey and the County of San Diego Health and Human Services Agency. SARS-CoV-2 = severe acute respiratory syndrome coronavirus 2; SES = socioeconomic status.

a The coefficient and standard error were multiplied by 1,000 for the ease of presentation.

† *p* < .1. *p < .05. **p < .01. ***p < .001 (two-tailed).

## Results

### New York City

The results for New York City can be found in Table 1, and map of the infection rates over the two clustering scores can be found in Figure 1. First, from the gross effects in the first column in the table, both black and Latino clustering are significantly related to the infection rate across zip codes. Moreover, these effects are substantively large. While the coefficients presented in the tables are the original coefficients, for discussion here, we convert them to standard deviation changes to compare across because the range of these variables is so different, especially the clustering scores. A standard deviation (1,561.47 for New York City) increase in black clustering (from an observed range of 0.24–7,789.5) is related to an increase of 23.42 in the rate of infections per 10,000 people in a zip code. This same figure is 39.16 for a standard deviation increase (1,305.22) in Latino clustering (which has a smaller observed range of 14–4,849.19). This pattern is evident in the map of New York City in Figure 1. The map shows the number of infections (as denoted by a proportional dot) over a choropleth map of the two clustering scores. From these maps, the heavily black and Latino sections of Harlem, the Bronx, Queens, and Brooklyn appear to have higher numbers of infections, while Manhattan has far fewer infections, despite a high population density in this area. Indeed, the variable for population density in this first model is significant and negative, meaning that density does not account for the relationship between racial clustering and infections, and that it further appears to be inversely related in the case of New York City. From the pseudo-*R*^2^ value, these factors alone account for 41 percent of the variation in differences across zip codes in the infection rate.

The next models include several covariates that have been theorized to be related to health in general, or to the case of the COVID-19 pandemic in particular. We expected to find that some of these variables would explain away the association between racial clustering and infections, especially those which have been shown to be salient to the current pandemic. However, in the case of Latino clustering, none of these groups of variables reduces the coefficient to nonsignificance, although some reduce the size of the effect substantially, including socioeconomic variables and occupation. In the case of black clustering, the coefficient is reduced to nonsignificance in two cases, the socioeconomic variables and occupation. In the case of socioeconomic variables, this seems to be largely driven by education, as the percent of the population with a bachelor’s degree or higher is significant and negative, meaning that the more educated the population in a zip code, the lower the infection rate. The size of this effect is large as well. For every standard deviation increase (21.73 percentage points) in the percent of people with a bachelor’s degree in a zip code, the infection rate drops by 45.98 infections per 10,000. In the case of the occupation variables, both percent sales and percent production are significant and positive, meaning that the higher percent of the zip code population that is employed in sales or production and transportation occupations, the higher the number of infections in that area. These are similarly large increases as well, with 14.10 and 18.99 more infections per 10,000 in a zip code for each standard deviation increase in the percent of population that works in sales (*SD* = 3.05) and production (*SD* = 4.5), respectively.

### Chicago

Turning to the results in Table 2 and Figure 2 for Chicago, the results are similar to the case of New York City. Again, in the first column with the gross effects of black and Latino clustering, both are significant and positive, indicating that a higher degree of concentration and clustering of these groups is related to a higher infection rate. The sizes of these are similarly large as in New York City, but Chicago is somewhat more segregated. For every one standard deviation increase (1,475.06 for Chicago) in black clustering (with an observed range of 0–8,086.17), the infection rate is expected to increase by 22.13 infections per 10,000. This same coefficient for Latino clustering (with a standard deviation of 712.52 and an observed range of 0–6,024.8) is 29.21 more infections per 10,000 people. In the case of Chicago, though, population density is not significant in either direction. This pattern is evident in the map of Figure 2 where infections are rather starkly clustered in the predominantly black South side of Chicago and the pockets of Latino clustering in the Western parts of the Chicago area, while the white suburbs of Chicago exhibit far lower rates of infection. From the pseudo-*R*^2^ value, these factors alone account for 49 percent of the variation in differences across zip codes in the infection rate.

With regard to the other variables, Chicago is again fairly similar to New York City in terms of significance and direction, and how they relate to the two clustering scores. Once again, Latino clustering is significant and rather robust across all model specifications. And, just like the case of New York City, the area-level socioeconomic variables reduce black clustering to nonsignificance (though not occupation in this case). Here, all of the socioeconomic variables are significant and in the expected direction—education as measured by percent college educated is negative, while percent in poverty and the unemployment rate are both positively related to a higher infection rate. Interestingly again, none of the more traditional public health measures related to infectious disease, such as density, age, household crowding, and commute, explain away the effect of segregation even though they are significant and in the expected direction in most cases.

### Houston

Next, we address the results for Houston in Table 3 and Figure 3. This set of results is different than in the case of New York City and Chicago. The variables included generally seem to do a worse job in explaining the variation in cases across zip codes in the Houston area. Fewer variables are significant, the effect sizes are smaller, and the pseudo-*R*^2^ values are far lower for all model specifications. From the map in Figure 3, there does not seem to be as strong of a pattern to the distribution of cases across space. As it relates to segregation, in the gross effects, both black and Latino clustering are significant and positive, meaning that a higher concentration and clustering of these two groups across space is related to a higher infection rate. These effect sizes are smaller, though, at the time of the study period, Houston had far fewer cases than cities like New York City (though these patterns have changed over the course of the pandemic so far). For every one standard deviation increase (764.30 for Houston) in black clustering (with an observed range of 3.94–4,684.6), the infection rate increases by 6.88 infections per 10,000 people, and this same figure is only 1.8 for a standard deviation increase (1,800.94) in Latino clustering (with an observed range of 47.1–7,894.7). However, these variables combined with population density only account for 26.2 percent of the variation in the infection rate across zip codes.

Moreover, as we add relevant variables to the model, we also observe a divergent trend from New York City and Chicago. In the case of Houston, the score for black segregation is never reduced to nonsignificance, and the size of the effect is only slightly reduced when accounting for socioeconomic status and commuting factors. The occupation variables are all nonsignificant as well and do not alter the size of the effect for black clustering. For Latino clustering, though, most of the other clusters of variables, with the exception of commuting factors, reduce the coefficient to nonsignificance. Moreover, only a few variables are significant across the various model specifications. In the model subsets, only percent in poverty, residential instability, and the percent of people who rely on public transportation are significant and positive (in the expected direction). A few more coefficients are significant in the full model, but the full model includes a variety of related types of variables, and there are clearly some suppression effects in the final model when comparing the reduced with the full models. Thus, in the case of Houston, the spatial patterning by segregation appears to be more consistent for black segregation but is explained away by several other covariates for Latino segregation.

### San Diego

Turning to the results for San Diego in Table 4 and Figure 4, we again observe a pattern that is different from New York City and Chicago. The most glaring difference is that black clustering is not significant for any model specification in the case of San Diego. However, the black population of San Diego is quite small at only 5 percent and is not particularly segregated as noted above, with the upper bound of the clustering score at 217.5. The results for the Latino clustering score, though, are strong and consistent across all model specifications. Latinos make up close to a third of the population and are much more segregated (an observed range of 33.1–6,454.6 for the Latino clustering score). The size of the effect is smaller than in the case of New York City or Chicago, but San Diego has had substantially fewer cases than any of the other cities studied here, especially for the first wave in May when the data were captured. Yet, the disparity by race is fairly large here. For the gross effects, a standard deviation increase (1,201.38 for San Diego) in Latino clustering is related to an increase in the infection rate by 10.81 infections per 10,000 people. The map in Figure 4 (with just the results for Latino clustering as black clustering is not significant) presents a clear indication of this pattern. The cases are strongly clustered in the non-coastal area of the county south of downtown and close to the U.S.-Mexico border, and in some of the eastern suburbs. This is also where there is the most clustering of the Latino population. The variables in the first model alone account for 46 percent of the variation in the infection rate by zip code, and that seems to be largely driven by the Latino clustering variable as the only significant variable in the model.

In the case of San Diego, the other variables included only minimally change the size of the effect of Latino clustering, even while some of them are significant and in the expected direction. The socioeconomic variables (in this case percent bachelor’s degree and the unemployment rate are both significant) and the household variables seem to do the best job of explaining the relationship between the clustering score and the outcome, but in both cases, the size of the coefficient is only reduced to 0.007, or an increase of 8.4 infections per 10,000 for each standard deviation increase (1,204.38) in the clustering score. Interestingly, for San Diego, which is the only location studied here on the border, the immigration score is significant and negative, meaning that as the percent of the foreign-born population increases, the infection rate decreases (after accounting for the concentration and clustering of Latinos).

## Discussion and Conclusions

The goal of this study is to examine the differences across neighborhoods in the distribution of SARS-CoV-2 infection rates to better understand how the current pandemic relates to broader patterns of urban and racial inequality. Specifically, we examine racial/ethnic residential segregation, as measured by residential clustering, which we argue could undergird the patterns of inequality in infection rates by race/ethnicity across the country. We demonstrate these patterns using four case cities across the United States: New York City, Chicago, Houston, and San Diego.

Overall, we find that racial/ethnic residential clustering is a rather strong predictor of infection rates across areas, though the patterns of this association vary somewhat by the group and location in question. In New York City and Chicago, two large, densely populated, and highly segregated American cities in the North, both black and Latino segregation, are strongly tied to SARS-CoV-2 infection rates. This provides support for Hypothesis 1. These two cities were both hit hard during the first wave of the virus during March and April of 2020. Moreover, in both of these cases, the only variables that reduced the association between the segregation variables and outcome were area-level indicators of socioeconomic status. Thus, while other covariates, which are more common in epidemiological studies of infection, seem to be related to infection rates, like age or housing crowding, the socioeconomic status variables seem to be the most salient for understanding the link between segregation and area-level infection rates of this particular virus. This provides only partial support for Hypothesis 2 in these two cases.

This finding is generally expected. New York City and Chicago are two of the most segregated cities in the United States, and this is fitting with the work in urban sociology on segregation more broadly that has demonstrated particularly acute consequences of segregation for the large, historically industrial cities of the Midwest and Northeast. Indeed, Chicago is the most-oft studied city in attempting to understand both the causes and consequences of segregation in urban sociology (Sampson 2012). Moreover, these cities both have well-developed public transportation systems and thereby a greater reliance on public transportation for work commutes. There is also a high degree of occupational segmentation by race in these areas as well. Early work on the pandemic has shown that black and Latino populations are more likely to be employed as essential workers, rely on public transportation, and less likely to be able to work from home (Hawkins 2020; Rogers et al. 2020; Selden and Berdahl 2020; Tai et al. 2021). In addition, although studies focusing on Latinos are limited, research has found a large concentration of Latinos in the food-service sector (Selden and Berdahl 2020) and in the medical field as personal care aides and medical assistants (Hawkins 2020). In addition, research demonstrates that black and Latino essential workers are less likely to be able to work from home than whites (Selden and Berdahl 2020). Overall, the higher probability of being an essential worker among blacks and Latinos may contribute to their higher COVID-19 infection rates as they work jobs where they interact closely with others. These findings fit well with the current state of literature.

The findings for Houston and San Diego diverge somewhat from these findings, but still highlight the importance of residential segregation in shaping health. In both locations, we find a strong association between the segregation scores and infection rates, though these associations are qualified. For Houston, we find that in the gross effects, both black and Latino segregation are related to a higher infection rate in the zip code, but this effect drops to nonsignificance when accounting for several other covariates in the case of Latino clustering, but remains consistent for black segregation. For San Diego, we find the opposite. The clustering score is only significant for Latino segregation, but as noted above, the black population of San Diego is small (5 percent of the total population), is not particularly clustered, and has low degree of global segregation using more traditional area-wide segregation measures, unlike what we observe in the three other cities, which each have black populations above 25 percent of the total population. This provides partial support for Hypotheses 1 and 2. For both of these locations, the infection rates at least for the first wave (in mid-May) were much lower across the board, and particularly in San Diego as California moved quickly and aggressively to implement measures to stop the spread of the illness. Both of these locations are also somewhat less segregated than New York City and Chicago, although they are hardly integrated. San Diego is also the only location directly on the U.S.-Mexico border and a sizable Latino immigrant population. This is fitting with previous work that generally finds that the Sunbelt cities of the South and West experience somewhat lower rates of segregation and fewer consequences as a result (Massey 2020). Yet, given this more conservative test, we still observe important patterns by segregation, and it is clear that racial/ethnic minority neighborhoods are bearing a disproportionate burden of this disease across the population.

Overall, these results track well with previous studies on racial/ethnic residential segregation and health outcomes that demonstrate notable disparities in health where racial/ethnic segregated communities and cities are more likely to experience poor health outcomes (Williams and Collins 2001; Yang, Park, and Matthews 2020). This includes the more limited work on infectious diseases specifically (Acevedo-Garcia 2000, 2001). However, the results presented here present an advance in this research as the work on infectious disease is much more limited compared with other health outcomes as is typical in modern health research, which is more focused on chronic disease (Acevedo-Garcia et al. 2003). Where these results diverge from the previous literature is that much of the extant work is highly focused on the case of black segregation, with more qualified or mixed effects for Latino segregation (Anderson and Fullerton 2014; Gibbons and Yang 2014). However, with the exception of Houston, the results for Latino segregation seem to be consistently stronger with larger effect sizes, and in most cases, the covariates do not explain away the association. Thus, there seems to be something unique about the current pandemic that produces a particularly strong association with Latino segregation, one that often appears to be even more consistent than in the case of black segregation.

As it relates to the literature on COVID-19 specifically, a growing body of work has begun to examine how racial/ethnic residential segregation contributes to racial disparities in COVID-19 transmission and deaths, and these results largely track with these early findings (Hawkins 2020; Khanijahani and Tomassoni 2021; Kim and Bostwick 2020; Millet et al. 2020; Rogers et al. 2020; Selden and Berdahl 2020; Tan et al. 2020; Yang et al. 2021; Yu et al. 2021). These studies display robust results that show that the features of racially segregated areas exacerbate the transmission and effects of the COVID-19 disease. However, these studies have limitations that we hope our analyses can address. First, many studies only examine segregation at the county level, which is a large geographic unit of analysis that cannot measure more neighborhood-level effects of segregation (Khanijahani and Tomassoni 2021; Millet et al. 2020; Tan et al. 2020; Yang et al. 2021; Yu et al. 2021). By using zip-code-level data, we are able to better account for these fine-grained dimensions of segregation and take into account both the concentration and clustering of racial/ethnic minorities. Next, several studies only use COVID-19 deaths in their analyses, which does not as accurately capture the mechanisms we hope to test (Khanijahani and Tomassoni 2021; Kim and Bostwick 2020; Yu et al. 2021). While the conditions of racially segregated areas may contribute to an increased probability of dying from COVID-19, we argue that looking at total infections better captures the prevalence and transmission of SARS-CoV-2 in these areas, as well as better captures how the risk factors in segregated areas affect the people at risk of infection, beyond mortality. Research on segregation and infectious disease generally looks at how segregated areas exacerbate the transmission of infectious diseases rather than risk of death from them (Acevedo-Garcia 2000, 2001). By looking at total SARS-CoV-2 infections rather than COVID-19 deaths, our study provides a better test of how segregated areas transmit COVID-19 to structurally disadvantaged groups and infect large amounts of people in resource-deprived spaces.

Despite this insight into the novel coronavirus pandemic, the study has limitations. First, as noted above, at this point in the pandemic, due to limitations and the decentralized way in which data have been reported across the United States, comparable data with more complete information about the affected populations are difficult to acquire at a small unit of analysis like the zip code. This reporting has occurred mostly at the level of the city or county, depending on how local public health jurisdictions are organized. This has meant that availability and data on testing has been quite different across place, making it difficult to compare across locations or to study the entire United States. We hope that over time, more data will become available or that a more unified federal testing and reporting apparatus will make these data more comparable for analytical purposes. Ideally, these data would include measures of not just the number of infections but also the number of tests, testing availability, and the infection rates broken down by key demographics. More unified, comparable measures of infection rates would allow for a more comprehensive analysis of the United States, as well as of whole metropolitan areas, as this best represents a more relevant region/area for analysis in terms of access to resources (such as testing and vaccines) and transportation systems. Future work should consider this important gap in our study. Similarly, with this analysis, it is hard to disentangle the effects of segregation from selection and composition effects. Because racial/ethnic minorities are more likely to contract the virus, when their numbers are grouped by residence, we would expect the rates by zip code to be higher. However, this is partially the point of our study, to demonstrate how segregation can cluster social problems in an area in a manner that would not exist if segregation were not present. Furthermore, as a check on this selection bias critique, for Chicago, we were able to run all of the same analyses using the infection rates by race and normalizing by the relative size of the group’s population in that area, and we found that black and Latino clustering scores were similarly related to the outcome. We were only able to do this for Chicago, though, as other locales did not provide their data in this fashion, and many locations had inconsistent and missing data for infections by race. Being able to conduct this analysis across the United States using race-specific infection rates would be optimal and an important consideration for future studies.

Furthermore, all of the independent variables come from the ACS and reflect people’s situations prior to the pandemic and not how their behavior may have changed in the pandemic. For example, we include a measure on the typical work commute on rates of public transportation use. While this can provide an indication of public transportation dependency in the area, actual use of these services likely declined in the pandemic with many individuals seeking out alternative means of transportation out of concerns for safety or working from home. Some recent work has employed clever techniques using cell phone data to track the mobility of individuals over time throughout the pandemic (Klein et al. 2020). However, these data are only available at an aggregated level of analysis, such as the metropolitan level. The study could also be improved with more independent variables on behaviors that are relevant to the current pandemic, such as mask wearing, mobility, public health orders, and compliance with local public health orders.

Overall, this study sheds light on the ongoing public health crisis and links patterns of segregation to the SARS-CoV-2 infection rate. From our preliminary evidence, it appears as though black and Latino segregated neighborhoods are suffering disproportionately from the COVID-19 pandemic in a manner that is not explained away by several other contextual variables. We find this pattern across four major U.S. cities, with some variation depending on the group and location in question. Furthermore, we find these patterns despite being a fairly conservative test using only infections from the first wave of the pandemic. We intentionally picked dates from the end of the first wave in the spring when most major U.S. cities were under lockdown orders of some sort and many businesses were closed. We anticipate that these findings would change over time and likely strengthen with the increased rate observed in many locales after the reopening of the economy. The study also has implications for public health intervention. While much of the coverage of the pandemic has highlighted racial disparities in outcomes, little work has demonstrated how the spatial elements of race relations are related to these trends. However, space and place play a strong role in interventions. For example, some news reporting has found disparities in where testing sites are located (*npr* 2020). If we know that racial/ethnic minority segregation is related to infection rates, local public health entities should more carefully consider the location of their sites for testing, treatment capacity, and vaccine allocations as transmission rates appear to be both raced and placed.

## Notes

1. We originally intended to study Los Angeles to capture the four largest cities in the United States, which also represent the four major Census regions. However, the data for Los Angeles were reported by city or “community” (known neighborhoods in LA), which encompass much larger areas, were not comparable with the other data for the other three cities, and could not be directly paired with the Census as the data do not include a standard identification code. For this reason, we chose the next largest West Coast city, San Diego.

2. Each of these represent slightly different geographies based on the public health reporting agencies of each locale. For New York City, the unit is the city level; for Chicago, it is the metropolitan area (at least the part contained within Illinois, not Indiana); and for Houston and San Diego, it comes from the core counties of those areas. While it would be ideal to include the entire metropolitan area for all four areas, as this best reflects the region where most interaction and travel are occurring, this is not possible due to data limitations. For example, in Houston, this would involve collection of data from nine different county health departments, some of which do not report data at the zip code level, and all of which do not collect and report their data in the same fashion. Thus, while metropolitan areas would be optimal, it was not possible for this analysis due to the decentralized way in which data on infections have been collected and reported throughout the pandemic.

3. As a check on our choice of measure, we also ran all of the same models using density scores, or percent black and percent Latino in a zip code, and the patterns were similar. Indeed, the substantive effect sizes were larger using this specification. We use these clustering scores as we think they better conceptually represent the extent of the geographic clustering of groups across space.

4. We understand that this does not include the full racial/ethnic diversity present in the United States. However, we include these two groups as being relevant to the pandemic and which have sufficiently large populations in our four case cities for analysis. The numbers from the Centers for Disease Control and Prevention (CDC 2020b) indicate that black, Latino, and Indigenous populations are suffering disproportionately from the virus. In the case of Indigenous populations, though, these groups make up too small of a percentage of the total population in our four case cities to analyze their population distribution and its relationship to viral transmission. Furthermore, we also exclude a score for Asian clustering as reports have not shown that the group is generally at greater risk for the illness (CDC 2020b). As a check on this choice, we also ran all of the same models using similar measures for whites and Asians. In all cases, these scores were either not significant, or they were significant and negative, meaning that segregation was related to a lower infection rate in the area. This was the case for Asian clustering in New York City and Chicago, and white clustering in Chicago and Houston. Due to multicollinearity, we could not include all four groups in a single model, so to include all groups, we would have to present several sets of models. For parsimony, we focus on the case of black and Latino clustering, which have been the focus of racial disparities in infection rates and deaths throughout the pandemic.
